# Origin and dynamics of oligodendrocytes in the developing brain: Implications for perinatal white matter injury

**DOI:** 10.1002/glia.23256

**Published:** 2017-11-14

**Authors:** Erik van Tilborg, Caroline G. M. de Theije, Maurik van Hal, Nienke Wagenaar, Linda S. de Vries, Manon J. Benders, David H. Rowitch, Cora H. Nijboer

**Affiliations:** ^1^ Laboratory of Neuroimmunology and Developmental Origins of Disease University Medical Center Utrecht, Utrecht University Utrecht The Netherlands; ^2^ Department of Neonatology University Medical Center Utrecht, Utrecht University Utrecht The Netherlands; ^3^ Department of Pediatrics, Eli and Edythe Broad Center for Stem Cell Research and Regeneration Medicine University of California, San Francisco San Francisco California; ^4^ Department of Paediatrics, Wellcome Trust‐MRC Stem Cell Institute University of Cambridge Cambridge United Kingdom

**Keywords:** brain development, myelination, oligodendrocyte precursor cells, preterm birth, white matter injury

## Abstract

Infants born prematurely are at high risk to develop white matter injury (WMI), due to exposure to hypoxic and/or inflammatory insults. Such perinatal insults negatively impact the maturation of oligodendrocytes (OLs), thereby causing deficits in myelination. To elucidate the precise pathophysiology underlying perinatal WMI, it is essential to fully understand the cellular mechanisms contributing to healthy/normal white matter development. OLs are responsible for myelination of axons. During brain development, OLs are generally derived from neuroepithelial zones, where neural stem cells committed to the OL lineage differentiate into OL precursor cells (OPCs). OPCs, in turn, develop into premyelinating OLs and finally mature into myelinating OLs. Recent studies revealed that OPCs develop in multiple waves and form potentially heterogeneous populations. Furthermore, it has been shown that myelination is a dynamic and plastic process with an excess of OPCs being generated and then abolished if not integrated into neural circuits. Myelination patterns between rodents and humans show high spatial and temporal similarity. Therefore, experimental studies on OL biology may provide novel insights into the pathophysiology of WMI in the preterm infant and offers new perspectives on potential treatments for these patients.

## 1 WHITE MATTER INJURY IN PRETERM INFANTS

Approximately 10% of all births occur prematurely, that is, before 37 weeks of gestation (Liu et al., 2012a, 2012b). Preterm infants spend the first weeks of life under suboptimal extra‐uterine conditions, during which they often encounter inflammatory and hypoxic insults due to immature organs, vascularization, and immune system (Volpe, Kinney, Jensen, & Rosenberg, 2011). Such insults can have severe consequences on brain development, leading to neurological problems later in life (Ancel et al., 2015; Stoll et al., 2015). The white matter in preterm infants is particularly vulnerable to injury due to crucial processes in white matter development taking place during the third trimester of pregnancy (Salmaso, Jablonska, Scafidi, Vaccarino, & Gallo, 2014; Volpe, 2009). Consequently, at present, the most commonly observed type of brain injury in preterm infants is white matter injury (WMI) (Back & Miller, 2014).

**Figure 1:**
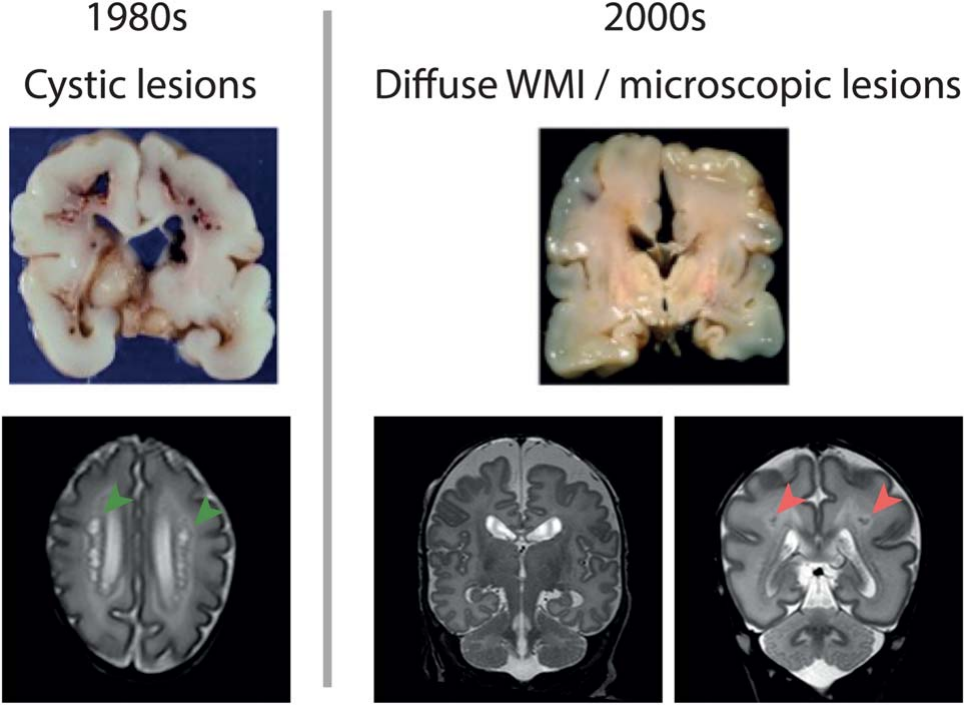
The clinical problem of perinatal white matter injury (WMI) has evolved over time. Upper panels: photographs of postmortem brain slices of preterm infants with WMI (published with permission from http://neuropathology-web.org/). Lower panels: T2‐weighted MR images of preterm infants with WMI. Left part of the figure: in the 1980s, cystic periventricular leukomalacia (cPVL) was often observed in preterm infants. cPVL is associated with large cystic lesions in the white matter clearly present at macroscopic postmortem tissue and at (transverse) MRI scan (T2 sequence) as indicated by the green arrowheads. cPVL leads to severe disabilities such as cerebral palsy. Right part of the figure: at present, diffuse WMI is most often associated with atrophy of white matter causing loss of brain volume (middle (coronal) MRI scan) and punctate white matter lesions (PWML) (right (coronal) MRI scan: red arrowheads). Diffuse types of WMI are mainly associated with impaired cognitive functioning later in life

Over the past decades, the clinical presentation of perinatal WMI has changed. In the 1980s, relatively large cystic lesions in the white matter were observed in 5%–10% of extremely preterm infants (born before a gestational age of 28 weeks) and this type of WMI was referred to as cystic periventricular leukomalacia (cPVL). cPVL is defined as necrosis forming cystic lesions localized in the deep periventricular white matter that are well visualized by cranial ultrasound, and by MRI (Khwaja & Volpe, 2008) (Figure 1). The cysts resolve over weeks to months, causing ex‐vacuo dilatation and periventricular glial scar formation as observed on MRI scans. cPVL is almost invariably related to severe impairment of neurological functioning, including serious disabilities such as cerebral palsy. Over the past decades, cPVL has become less common and the incidence has decreased to below 1% in some centers (Gano et al., 2015; Hamrick et al., 2004; van Haastert et al., 2011). Presently, the term “WMI” is increasingly being used and rarely refers to cystic WMI, but rather refers to diffuse WMI (Back, 2006; Woodward, Anderson, Austin, Howard, & Inder, 2006). In diffuse WMI instead of macroscopic cysts, microscopic cysts (not detected by ultrasound or MRI) develop and evolve into glial scars with marked astrogliosis and microgliosis over the course of several weeks (Volpe, 2009). Furthermore, diffuse WMI is associated with reduced total white matter volumes, thinning of important white matter tracts, ventriculomegaly and altered white matter microstructure as measured by diffusion tensor imaging (Alexandrou et al., 2014; Glass et al., 2008; Mwaniki, Atieno, Lawn, & Newton, 2012; Rutherford et al., 2010; Shankaran et al., 2006; van Vliet, de Kieviet, Oosterlaan, & van Elburg, 2013). Besides atrophy of white matter tracts and diffuse changes in signal intensity in the white matter on MRI, punctate white matter lesions (PWML) are observed in the periventricular white matter in as many as 20% of extremely preterm infants (Figure 1). PWML appear as focal signal intensity changes on conventional MRI and as restricted diffusion when the MRI is performed within a week after the presumed insult suggestive of ischemic injury (Kersbergen et al., 2014). Compared to cPVL, diffuse WMI and PWML are related to a milder degree of neurological impairment affecting mostly cognitive functioning and increased risk of psychological disorders later in life (Back & Miller, 2014; Guo et al., 2017; Keunen et al., 2017; Woodward, Clark, Bora, & Inder, 2012).

Based on pathology studies using human postmortem brain tissue, the stressful environment that preterm infants reside in during the first weeks of life is thought to negatively affect the developmental programming of oligodendrocytes (OLs), the cells responsible for myelination (Billiards et al., 2008; Buser et al., 2012; Verney et al., 2012). An arrest in OL maturation due to perinatal insults causes myelination deficits associated with perinatal WMI (Volpe et al., 2011). In order to elucidate the precise pathophysiology underlying perinatal WMI, it is crucial to fully understand the mechanisms through which OLs develop and achieve myelination during normal white matter development. In the next sections, we will describe fundamental aspects contributing to white matter development starting with the generation of oligodendrocyte precursor cells (OPCs), followed by the temporal and spatial patterns through which these cells populate and eventually myelinate the central nervous system (CNS). Furthermore, heterogeneity within the OL lineage is discussed. Finally, we translate these findings to the human situation by discussing similarities and differences between myelination patterns in rodents versus humans and we highlight the possible implications for WMI in preterm infants.

## 2 OPC GENERATION

**Figure 2:**
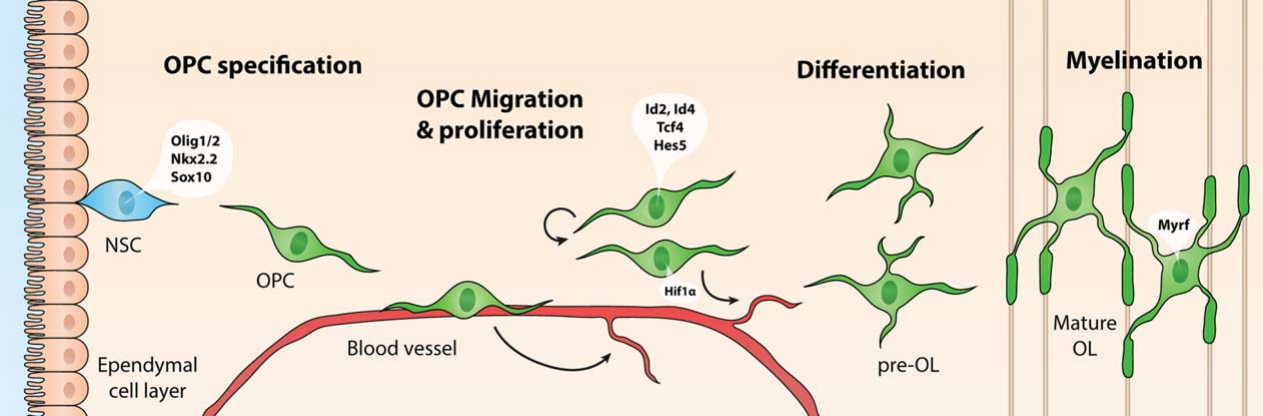
Schematic representation of oligodendrocyte (OL) development and transcription factors that contribute to OL lineage progression at different developmental stages. OL precursor cells (OPCs) originate from neuroepithelial zones surrounding the ventricles, where neural stem cells (NSCs) differentiate into (OPCs) under the influence of OL‐specific transcription factors Olig1/2, Nkx2.2, and Sox10. OPCs migrate toward an appropriate site via blood vessels, while at the same time promoting angiogenesis in a HIF1α‐dependent manner, in areas requiring more oxygen. At their final destination, OPCs proliferate to expand the pool of OPCs, under the regulation of transcription factors such as Id2, Id4, Tcf4, and Hes5. When proliferation is inhibited, OLs differentiate into premyelinating OLs (pre‐OLs), and finally into mature OLs that enwrap neuronal axons with myelin sheaths, under the influence of, for example, Myrf

The white matter contains myelinated axons that allow communication between distant brain regions. Myelin sheaths surrounding these axons are essential for proper brain connectivity, as myelination enables rapid and efficient propagation of action potentials, and provides protection and trophic support to axons (Funfschilling et al., 2012; Saab et al., 2016). In the central nervous system (CNS), oligodendrocytes (OLs) are responsible for the production of myelin. Since first being described by Pío del Río Hortega in 1921, much research has been done into OL biology and the developmental regulation and functions of these cells (Bergles & Richardson, 2015; Perez‐Cerda, Sanchez‐Gomez, & Matute, 2015). OLs originate from neural stem cell (NSC)‐derived oligodendrocyte precursor cells (OPCs) that differentiate into immature premyelinating OLs (pre‐OLs) and finally differentiate into mature OLs that contact neuronal axons and start the production of myelin (Figure 2) (Emery, 2010).

**Figure 3:**
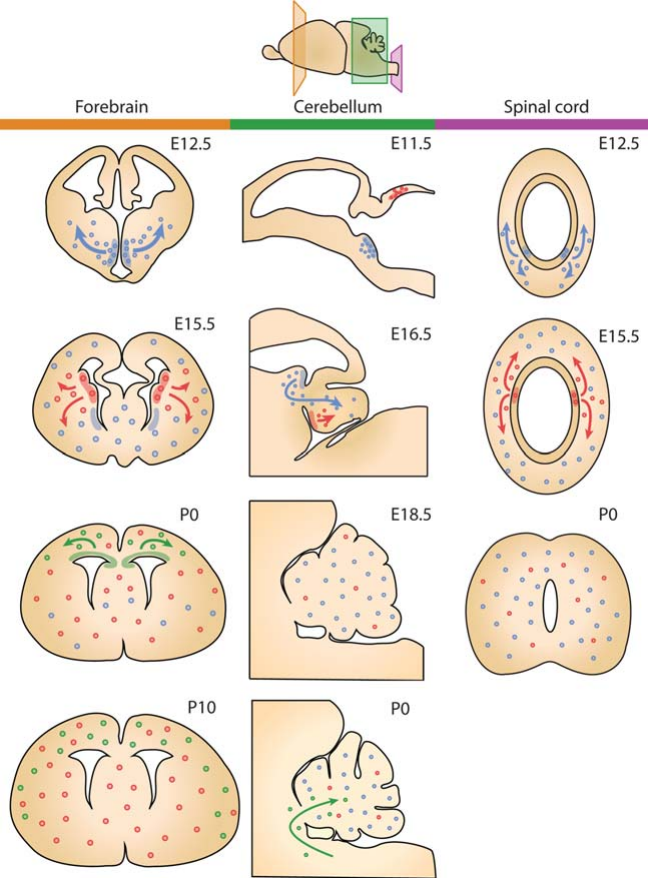
Schematic representation of how different waves of OPC generation populate different regions of the CNS throughout development (panels: left/orange: forebrain; middle/green: cerebellum; right/purple: spinal cord). OPCs originating from different niches are represented by differently colored dots (blue, red, and green) (based on data by Fogarty, Richardson, & Kessaris, 2005; Grimaldi, Parras, Guillemot, Rossi, & Wassef, 2009; Hashimoto et al., 2016; Kessaris et al., 2006; Ravanelli & Appel, 2015; Vallstedt, Klos, & Ericson, 2005)

Throughout the CNS, OPCs generally originate from neuroepithelial zones surrounding the ventricles. Here, proliferative NSCs commit to the OL lineage under the influence of transcription factors such as Olig1, Olig2, Nkx2.2, and Sox10 (Figure 2) (Emery, 2010). During development, OPCs can also derive from radial glial cells (Casper & McCarthy, 2006; Merkle, Tramontin, Garcia‐Verdugo, & Alvarez‐Buylla, 2004). The origin and dispersion of OPCs have been extensively studied in the rodent CNS, particularly in the forebrain, the cerebellum and the spinal cord. Interestingly, OPCs are generated in multiple waves, starting with a ventral wave, which shifts toward a more dorsal origin during the second wave (Figure 3) (Fancy et al., 2009; Kessaris et al., 2006).

There are also spatial differences in OPC development. In the murine forebrain OPCs originate from the subventricular zone (SVZ) in a ventral wave from the medial ganglionic eminence and the anterior entopeduncular area at embryonic day E12.5 (Figure 3, left panel, blue cells). Gradually, the stream of OPC generation moves dorsally with OPCs being produced from the lateral and caudal ganglionic eminences by the age of E15.5 (Figure 3, left panel, red cells). At birth (E21/P0), a wave of OPCs arises from the dorsal SVZ into the cortex (Kessaris et al., 2006; Rowitch & Kriegstein, 2010) (Figure 3, left panel, green cells). Remarkably, tracing studies revealed that OPCs derived from the first ventral wave are almost completely replaced postnatally, and that the complete population of OPCs in the forebrain at postnatal day P10 is derived from the medial and dorsal streams (Kessaris et al., 2006). However, when generation of OPCs during the second or third wave is disturbed, OPCs from the ventral wave do survive and differentiate to eventually myelinate the forebrain (Kessaris et al., 2006). These findings indicate that a competitive mechanism exists, creating a flexible system with the first, ventral wave acting as “backup” in case later waves are impaired. At P10, the OPCs derived from the medial wave evenly populate the entire forebrain, whereas the destination of OPCs from the dorsal stream is restricted to cortical areas (Kessaris et al., 2006).

Cerebellar OPCs have been shown to mostly originate from extracerebellar brain regions (Grimaldi, Parras, Guillemot, Rossi, & Wassef, 2009). More specifically, at E11.5, OPCs start to arise from the metencephalic ventral rhombomere 1 region and migrate toward the cerebellum where the first OPCs arrive at E16.5 (Figure 3, middle panel, blue cells). At E18.5, a large population of OPCs has reached the cerebellum, where these cells likely proliferate to further expand the cerebellar population of OPCs (Hashimoto et al., 2016). A second stream of OPCs is generated locally from the cerebellar ventricular zone (Figure 3, middle panel, red cells), but these cells comprise only 6% of the cerebellar OPC population at E18.5 (Hashimoto et al., 2016). After birth, neuroepithelial regions surrounding the fourth ventricle likely continue to produce OPCs that migrate toward the cerebellum (Reynolds & Wilkin, 1988; L. Zhang & Goldman, 1996) (Figure 3, middle panel, green cells).

In the spinal cord, the first OPCs arise from the ventrally located premotor neuron (pMN) domain, which starts to generate OPCs after E12.5 (Ravanelli & Appel, 2015; Richardson et al., 2000) (Figure 3, right panel, blue cells). At E15.5, a later wave of OPCs is generated from more dorsal precursor domains of the spinal cord (Fogarty, Richardson, & Kessaris, 2005; Vallstedt, Klos, & Ericson, 2005) (Figure 3, right panel, red cells). Spinal cord OPCs generated during the first ventral wave rapidly populate the whole plane of the spinal cord. OPCs that originate from the dorsal wave at E15.5 also distribute evenly across the spinal cord, but by the time of birth dorsally derived OPCs make up only 10%–20% of the OPCs and the majority of OPCs being derived from the ventral stream (Fogarty, Richardson, & Kessaris, 2005; Vallstedt, Klos, & Ericson, 2005).

The origin of OPCs in deep brain structures has not been studied extensively. Probably, OPCs in these regions are similarly derived from neuroepithelial zones surrounding the ventricles, from where they migrate toward the appropriate location and proliferate to expand the OPC population. For example, in the hypothalamus, OPCs are derived from a neuroepithelial niche surrounding the third ventricle from E13.5 onward with a peak at E17.5 (Marsters et al., 2016). Also, it has recently been demonstrated that OPCs generated at E12.5 in the preoptic area gradually migrate via the optic chiasm to eventually populate and myelinate the optic nerve (Ono et al., 2017).

## 3 OPC MIGRATION, PROLIFERATION, AND DIFFERENTIATION

### 3.1 OPC migration

OPCs contain growth‐cone like structures that sense numerous chemotactic cues to guide them toward their destination (Simpson & Armstrong, 1999). A wide variety of signaling molecules have been implicated in regulating OPC migration. For instance, spatial gradients of bone morphogenic proteins (BMPs), Sonic hedgehog (Shh) and Wnt proteins determine the direction of migrating OPCs. For example, dorsally secreted BMPs repel ventrally derived OPCs, thereby guiding them toward more ventral brain areas (Choe, Huynh, & Pleasure, 2014). In addition, different types of local cues, for example, growth factors, extracellular matrix proteins, axon guidance molecules and neuronal activity can affect OPC migration as well (de Castro & Bribian, 2005; de Castro, Bribian, & Ortega, 2013). Examples of growth factors that promote OPC migration include platelet‐derived growth factor (PDGF), vascular endothelial growth factor (VEGF), fibroblast growth factor (FGF) and hepatocyte growth factor (HGF) (Bribian, Barallobre, Soussi‐Yanicostas, & de Castro, 2006; Hayakawa et al., 2011, 2012; Milner et al., 1997; Murcia‐Belmonte, Medina‐Rodriguez, Bribian, de Castro, & Esteban, 2014; Yan & Rivkees, 2002). Furthermore, extracellular matrix components such as laminin, fibronectin, vitronectin, anosmin‐1, and tenascin‐C have been shown to stimulate migration of OPCs (Bribian et al., 2008; Garcion, Faissner, & ffrench‐Constant, 2001; Milner, Edwards, Streuli, & Ffrench‐Constant, 1996; Murcia‐Belmonte et al., 2016). Moreover, various factors associated with axon guidance, for example, neural cell adhesion molecules, semaphorins, netrin‐1 and the chemokine CXCL1, guide migrating OPCs by attraction or repulsion (Okada, Tominaga, Horiuchi, & Tomooka, 2007; Spassky et al., 2002; Tsai et al., 2002; Zhang, Vutskits, Calaora, Durbec, & Kiss, 2004). Interestingly, it has been shown that neural activation mediates OPC migration (Mangin, Li, Scafidi, & Gallo, 2012). By acting on AMPA receptors glutamate enables the formation of an AMPA/α_v_ integrin/proteolipid protein complex, which promotes motility. In addition, glutamate promotes OPC migration via NMDA receptors by stimulating expression of the polysialic acid‐neural cell adhesion molecule and by activating the Tiam1/Rac1/ERK signaling pathway (Gallo et al., 1996; Gudz, Komuro, & Macklin, 2006; C. Wang et al., 1996; Xiao et al., 2013; Yuan, Eisen, McBain, & Gallo, 1998).

In addition to local and neuronal cues, Tsai et al. (2016) recently demonstrated that proper brain vascularization is crucial for OPC migration. More specifically, it was shown that OPCs migrate by “crawling” along blood vessels (Figure 2), and that OPCs can also “jump” from one blood vessel to another (Tsai et al., 2016). Wnt‐mediated expression of the chemokine receptor CXCR4 on OPCs enables the coupling to blood vessels expressing the CXCR4 ligand SDF1 (CXCL12). Interestingly, increased Wnt signaling caused clustering of OPCs surrounding the vasculature (Tsai et al., 2016). Earlier reports demonstrated that OPCs can promote angiogenesis by monitoring oxygen tension through hypoxia‐inducible factor (HIF) signaling and by secreting Wnt7a/b in response to low oxygen levels, thereby promoting angiogenesis. Presumably, by doing this, OPCs ensure oxygen supply during myelination which requires high oxygen consumption (Yuen et al., 2014). Additionally, active interactions between pericytes and OPCs have been implicated in maintaining blood–brain barrier integrity (Maki et al., 2015; Seo et al., 2014). Collectively, these data highlight the importance of reciprocal interactions between OPCs and the vasculature (Figure 2).

### 3.2 Proliferation and differentiation

Once OPCs reach their destination, the population is expanded by proliferation. OPCs are highly proliferative cells that continue to divide until a homeostatic balance in the number of OPCs is achieved (Hughes, Kang, Fukaya, & Bergles, 2013). If this balance is disturbed due to cell death, differentiation or migration, local OPCs are triggered to proliferate to maintain a consistent pool of OPCs (Hughes, Kang, Fukaya, & Bergles, 2013). During development, OPC expansion is actively stimulated by extracellular signals that promote proliferation, but at the same time inhibit differentiation (for a list of regulatory pathways, see Table 1). One important signal is PDGF, which has been shown to potently drive OPC proliferation (Calver et al., 1998; Richardson, Pringle, Mosley, Westermark, & Dubois‐Dalcq, 1988). Other examples of signals that inhibit OPC differentiation include Notch and Wnt (Fancy et al., 2011; Genoud et al., 2002; Givogri et al., 2002; Guo et al., 2015; Kremer, Aktas, Hartung, & Kury, 2011; S. Wang et al., 1998). Downstream of these extracellular signaling molecules, transcription factors such as inhibitor of differentiation (Id)2, Id4 and Hes5 are responsible for preventing premature differentiation of OPCs. Downregulation of extracellular differentiation inhibitors during development relieves the “inhibition to differentiate,” for instance by promoting expression of microRNAs that prevent transcription of differentiation inhibitors (Dugas et al., 2010; Zhao et al., 2010). The disinhibition allows OPCs to differentiate into pre‐OLs and finally into mature, myelinating OLs under the influence of differentiation promoting transcription factors like myelin regulatory factor (Myrf) (Bujalka et al., 2013). As OLs mature, they contact neuronal axons and begin the expression of a large amount of myelin genes, including myelin‐associated glycoprotein (MAG), myelin oligodendrocyte glycoprotein (MOG), myelin basic protein (MBP) and myelin proteolipid protein (PLP), to start the assembly of myelin sheaths enwrapping axons (Nawaz et al., 2015). All in all, OL maturation and subsequent myelin assembly are tightly regulated processes that have been widely described earlier, but the exact mechanisms are beyond the scope of this review (for excellent reviews, see Emery, 2010; He & Lu, 2013; Mitew et al., 2014; Simons & Nave, 2015). Interestingly, throughout adulthood OPCs remain present, are required for myelin maintenance, and can be recruited to drive remyelination in case of injury to the myelin sheaths (Gautier et al., 2015). In adult mice, OPCs make up 8%–9% of the cell population in the white matter and 2%–3% of the population in the gray matter (de Castro et al., 2013; Dimou, Simon, Kirchhoff, Takebayashi, & Gotz, 2008).

**Table 1:** Regulators of OPC differentiation are promising therapeutic targets to promote white matter development in perinatal WMI

Pathway	Promote/inhibit OPC differentiation	References	Potential clinical intervention
BMP4 signaling	Inhibitor	Dizon et al., 2011; Reid et al., 2012; See et al., 2004	BMP4 inhibition; noggin
Endothelin 2	Promotor	Yuen et al., 2013	Endothelin receptor agonists
GABAergic signaling	Promotor (conflicting data)	Hamilton et al., 2017; Zonouzi et al., 2015	Antiepileptic drugs, e.g., tiagabine, vigabatrin
Hyaluronan/CD44 signaling	Inhibitor	Back et al., 2005; Buser et al., 2012; Hagen et al., 2014	CD44 inhibition; hyaluronidase
IGF1 signaling	Promotor	Cai et al., 2011; Pang et al., 2007; Pang et al., 2010a; Ye et al. 2007	IGF1 administration; cell based therapy
JAK/STAT signaling	Inhibitor	Raymond et al., 2011	JAK/STAT inhibition
JNK signaling	Inhibitor	Wang et al., 2012; Wang et al., 2014	JNK inhibition
Muscarinic acetylcholine signaling	Inhibitor	Deshmukh et al., 2013; Franklin 2015; Mei et al., 2014	Muscarinic acetylcholine inhibition; benztropine; clemastine
Notch signaling	Inhibitor	Scafidi et al., 2014; Wang et al., 1998	Notch inhibition; EGF
PDGF signaling	Inhibitor	Calver et al., 1998; Richardson et al., 1988	PDGF inhibition
Potassium signaling	Promotor	Fogal, McClaskey, Yan, Yan, & Rivkees, 2010; Zhu, Wendler, Shi, & Rivkees, 2014	K_ATP_ agonists; diazoxide
Pro‐inflammatory cytokines	Inhibitor	Favrais et al., 2011; Miron et al., 2013; Pang et al., 2010b; Taylor et al., 2010	Anti‐inflammatory treatments; activin A
Prostaglandin E2	Inhibitor	Gano et al., 2015; Shiow et al., 2017	Cyclooxygenase inhibition; indomethacin
Retinoid X receptor γ (RXR‐γ)	Promotor	Franklin 2015; Huang et al., 2011	RXR‐γ agonists; 9‐cis‐retinoic acid
Sirt1	Inhibitor	Jablonska et al., 2016	Class III HDAC inhibitors; sirtinol
Wnt/β‐catenin signaling	Inhibitor	Fancy et al., 2009, 2011; Lee et al., 2015a, 2015b	Wnt inhibition; Apcdd1 stimulation

## 4 OPC HETEROGENEITY

Over recent years, it has become clear that OPCs and OLs throughout the brain are not merely a homogeneous population of cells, but that different OPCs can express different markers and exert different functions. It has been proposed that different OPC subtypes can be classified based on various aspects such as their developmental stage, their origin, the expression of specific receptors/ion channels, or the spatial environment they reside in, which will be explained in more detail below.

### 4.1 Classification based on developmental stage

To explore heterogeneity in the OL lineage in detail, Marques et al. (2016) performed single‐cell sequencing on OPCs and OLs from various brain areas of juvenile and adult mice. Based on clustering of gene expression profiles, their data indicate that during the transformation from OPCs to mature OLs, cells progress through a strict program of six distinct developmental stages before maturing into myelinating OLs. Furthermore, they showed that based on their gene expression profile, during their final developmental stage mature OLs can be clustered into six additional subsets, indicating that also mature OLs eventually form a heterogeneous population (Marques et al., 2016). However, it should be noted that such a distinction may not necessarily reflect intrinsic OL differences as many transcripts that define the mature OL subsets are primarily neuronal; therefore, these interesting findings should be further validated to exclude the possibility of potential bias by environmental RNA.

### 4.2 Classification based on site of generation

As described in Section 2, during development, OPCs arise from different brain areas. This raises the question whether OPCs from different origins represent specific OPC subpopulations with distinct functional features (Ornelas et al., 2016). Kessaris et al. (2006) revealed that OPCs derived from the three successive waves depicted in Figure 3 are characterized by different transcription factors. Whereas the OPCs from the ventral stream express transcription factor Nkx2.1, OPCs from the medial and dorsal waves express transcription factors Gsh2 and Emx1, respectively (Kessaris et al., 2006). As OPCs derived from distinct waves possess intrinsically different genetic profiles, they may also exert functional differences. Indeed, depending on their origin OPCs have been shown to respond differently to certain stimuli. Whereas the generation and specification of ventrally derived OPCs depend on Shh signaling, generation of dorsal OPCs has been shown to be independent of Shh (Cai et al., 2005; Chandran et al., 2003; Nery, Wichterle, & Fishell, 2001). Additionally, Ortega et al. (2012) showed that migration of the first wave of OPCs, which populate the optic nerve, is dependent on signaling of the epidermal growth factor (EGF)‐related ligand neuregulin‐1 via its receptor ErbB4. However, migration of OPCs derived from later waves is not affected by neuregulin‐1, further indicating that OPCs from distinct waves show differential sensitivity to stimuli (Ortega et al., 2012). Moreover, Crawford et al. (2016) showed that in the mouse spinal cord, dorsally derived OPCs have a greater capacity to contribute to remyelination compared to ventrally derived OPCs. However, dorsal OPCs show a higher susceptibility to age‐related functional impairments (Crawford, Tripathi, Richardson, & Franklin, 2016). Together, these studies indicate that different OPC subpopulations do exist and that functionality of OPCs may partly depend on their developmental origin.

### 4.3 Classification based on expression of receptors/ion channels

Differences between OPC populations may not only be explained by their origin and the genetic factors that drive their specification, but also by the expression of specific proteins, for example, voltage‐gated ion channels (Clarke et al., 2012; Karadottir, Hamilton, Bakiri, & Attwell, 2008). More specifically, OPCs that engage in glutamatergic signaling and show voltage‐dependent depolarization have been shown to be more vulnerable to ischemic injury (Karadottir et al., 2008). Additionally, Vigano et al. (2016) identified a subpopulation of OPCs expressing G‐protein‐coupled receptor (GPR)17 present throughout the brain. Activation of GPR17 has been shown to negatively regulate OPC differentiation (Chen et al., 2009; Fumagalli et al., 2011). Indeed, GPR17+ OPCs in adult mice do not differentiate under normal circumstances, but have the ability to differentiate and start myelination in case of injury to other GPR17 negative OPCs, creating a reserve pool of OPCs that can compensate for OPC loss in the event of pathological situations (Bonfanti et al., 2017; Vigano et al., 2016). These GPR17‐expressing OPCs may represent a specific subpopulation of OPCs involved in remyelination. Whether OPCs with similar protein expression profiles (i.e., expression of ion channels, GPR17) share similar origins is currently unknown, but is an interesting issue for further research.

### 4.4 Classification based on location

Besides their developmental origin, the location that OPCs and OLs eventually reside in may also affect their functionality. This is illustrated by several differences that have been observed between OLs in the gray and white matter. For instance, in the spinal cord white matter OLs show altered localization of gap‐junction protein connexin‐32, decreased cell‐cell coupling and less susceptibility to metabolic disturbances compared to gray matter OLs (Bauer et al., 2002; Pastor, Kremer, Moller, Kettenmann, & Dermietzel, 1998). Furthermore, OLs in the gray matter have a lower differentiation capacity and their iron homeostasis is fully dependent on the ferroxidase hephaestin, whereas in the white matter loss of hephaestin can be compensated for by the ferroxidase ceruloplasmin (Dimou et al., 2008; Schulz, Vulpe, Harris, & David, 2011). It could be speculated that such region‐dependent differences are mediated by adaptations of OLs to their environment by reacting to local signaling molecules.

### 4.5 Intrinsic heterogeneity versus environmentally determined differences

As mentioned above, OPCs from distinct developmental origins are driven by distinct transcription factors. These diverse transcriptional profiles may account for functional differences between various subtypes of OPCs. However, based on data obtained from single‐cell RNA sequencing it might be more plausible that OPCs from different origins converge into a transcriptionally homogeneous pool of OPCs (Marques et al., 2016). As OPCs migrate and further develop, they may adapt their gene expression profile to the specific needs in the environment they reside in. In that case, OPC heterogeneity is mainly determined by environmental factors. To which extent various OPC subtypes are functionally interchangeable should be further elucidated in the coming years.

To summarize, more and more evidence indicates that OPCs and OLs form heterogeneous cell populations with differences in functions and differentiation capacities that are driven by both intrinsic and extrinsic factors. Likely, the origin and gene expression profile drive OLs to migrate and mature in a specific way that can be affected by local signaling molecules, eventually resulting in differences in expression of specific proteins like receptors (e.g., GPR17) and ion channels. Exactly how differences in origin, location and gene expression profile between OPC populations affect their functions should be further elucidated.

## 5 RODENT VERSUS HUMAN OL BIOLOGY

**Figure 4:**
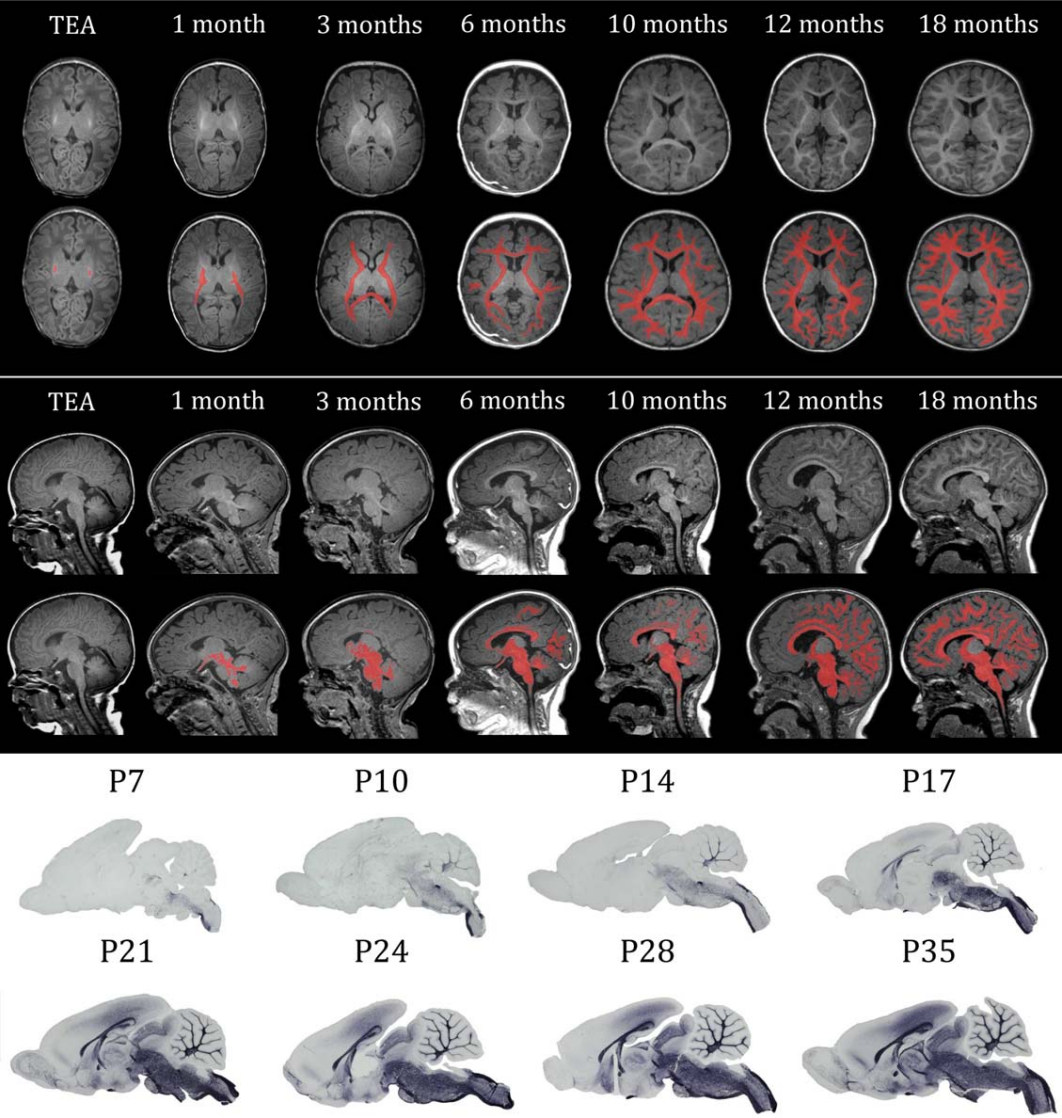
Overview of myelination throughout development from term‐equivalent age (TEA) to 18 months in human infants and from postnatal day (P)7 to P35 in rats. The upper panel shows transverse sections of T1‐weighted MRI scans at different ages, in the lower sections the myelinated white matter is manually colorized (red). The middle panel shows the sagittal sections of T1‐weighted MRI scans at different ages, in the lower sections the myelinated white matter is manually colorized (red). The lower panel shows sagittal sections of rat brains at different ages, stained for myelin basic protein (MBP), a myelin marker. The gross spatio‐temporal pattern of myelination in humans shows high resemblance with that of rodents

**Figure 5:**
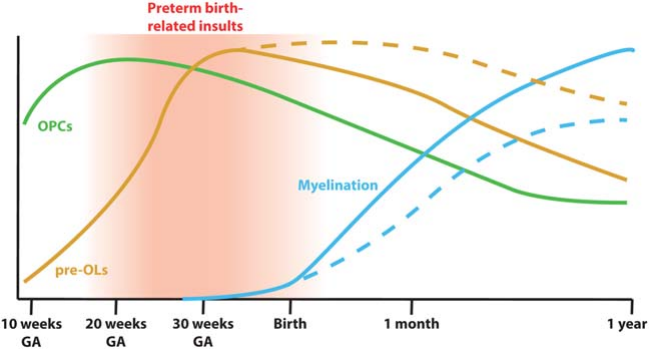
Developmental progression of OPCs, pre‐OLs, and myelination. The population of OPCs is expanded between 15 and 20 weeks gestational age (GA) (green line). Pre‐OLs peak between 30 and 40 weeks GA (orange line). Myelination starts before birth, but mostly occurs during the first year of life and continues for several decades (blue line). Children born prematurely are exposed to perinatal insults during the peak of pre‐OLs (red window), which hamper their ability to differentiate into myelinating OLs resulting in increased numbers of pre‐OLs and reduced myelination as observed in diffuse WMI (dashed orange and blue lines) (based on data by Back et al., 2001; Buser et al., 2012)

Human white matter development is a time‐consuming process. Although the axonal bundles that form the basis of the white matter are mostly in place before the third trimester of pregnancy, myelination of the first axons does not start until 30 weeks gestational age (GA) and predominantly occurs postnatally (Huppi et al., 1998; Inder & Huppi, 2000; Semple, Blomgren, Gimlin, Ferriero, & Noble‐Haeusslein, 2013) (Figures 4 and 5). The number of OLs in the human white matter increases until the age of 5 years and remains stable after this age, as determined by nuclear bomb test‐derived carbon (^14^C) dating (Yeung et al., 2014). Most myelination occurs during the first year of life (Aubert‐Broche, Fonov, Leppert, Pike, & Collins, 2008), but myelinated white matter volumes, particularly of frontal brain regions, keep growing until the age of ∼40 years (Bartzokis et al., 2001). Most knowledge regarding the generation and maturation of OPCs that myelinate the white matter is derived from rodent studies, as described above. To which extent findings from rodent studies can be extrapolated to the human situation is an important issue for translational purposes. Below, we discuss several similarities and differences in rodent versus human white matter development.

### 5.1 Similarities between rodent and human myelination

Careful analysis of human fetal brain tissue revealed that the first OPCs emerge in the developing brain at 10 weeks GA, followed by an expansion of the population before mid‐gestation (15–20 weeks GA) (Jakovcevski, Filipovic, Mo, Rakic, & Zecevic, 2009; Jakovcevski & Zecevic, 2005a; Rivkin et al., 1995). Several findings indicate that the ventral‐to‐dorsal routes of OPC generation are conserved in humans (Jakovcevski et al., 2009; Jakovcevski & Zecevic, 2005b). Similar to the rodent situation, OPCs in the human forebrain are derived from the lateral‐medial ganglionic eminence and the SVZ from where they migrate toward the cortical plate (Jakovcevski & Zecevic, 2005b; Rakic & Zecevic, 2003). In addition, transcription factors associated with ventrally derived OPCs in rodents, Dlx2 and Nkx2.1, have also been observed in human cortical OPCs. A subpopulation of OPCs that does not express these transcription factors has also been identified, but whether these cells express factors associated with a dorsal origin was not investigated (Rakic & Zecevic, 2003). Further evidence of OPC heterogeneity in the human brain comes from Leong et al. (2014), who demonstrated the presence of different OPC subtypes based on marker expression (A2B5, O4, and MOG) in both fetal and adult brain tissue using fluorescence activated cell sorting. Besides differential expression of OPC markers, it was shown that the microRNA expression profile differs between fetal and adult OPCs (Leong et al., 2014). Together, these observations raise the possibility that the human brain also contains different subpopulations of OPCs, originating from different brain areas, expressing different transcription factors and showing diverse phenotypes.

At an anatomical level, myelination patterns also show a high degree of similarity between rodents and humans, as illustrated in Figure 4. In the human brain, the caudal‐to‐rostral order of myelination is conserved with early myelination of the brain stem, midbrain and cerebellum during the first three months of life. Later, myelination of the telencephalon occurs in a caudal‐to rostral manner starting from occipital cortical regions toward, lastly, the prefrontal cortex (Inder & Huppi, 2000; Jakovcevski et al., 2009; Jakovcevski & Zecevic, 2005b).

### 5.2 Differences between rodent and human myelination

Although myelination throughout development is rather conserved between species, differences between rodent and human OL biology have also been described. One obvious difference is brain size; compared to rodents, humans possess a 3000× larger brain volume and the relative proportion of white matter in the brain has greatly increased throughout evolution (Semple et al., 2013; Zhang & Sejnowski, 2000). Whereas myelin proteins have been shown to be extremely long‐lived (Savas, Toyama, Xu, Yates, & Hetzer, 2012), several reports indicate that a relatively high turnover of OLs is crucial for myelin maintenance in rodents (Gibson et al., 2014; Young et al., 2013). In contrast, human OLs have been shown to have a low annual turnover rate of 0.3%, indicating that human myelinating OLs survive for a long time (Yeung et al., 2014). These findings indicate that the dynamics by which the pool of OL is maintained may differ between species. However, it should be noted that OPC maintenance has been investigated in much less detail in humans, compared to rodents. Of note, the observed differences may also be explained by regional differences, that is, whereas in some brain regions OL turnover is low, other brain regions may require higher rates of OL turnover.

Moreover, several differences between rodent and human OLs have been reported at a cellular level. For instance, OPCs cultured from human fetal brain tissue show reduced responsiveness to the mitogens bFGF and CXCL1 compared to rodent OPCs (Filipovic & Zecevic, 2008; Wilson, Onischke, & Raine, 2003). These data indicate that not necessarily all findings in rodent OPCs can be straightforwardly translated to the human situation. Over the past years, the technology of producing human OPCs and mature OLs from induced pluripotent stem cells (iPSCs) has become available (Douvaras et al., 2014; S. Wang et al., 2013). Human iPSC‐derived OPCs may be used to more specifically assess the biology of human OLs in vitro and to investigate to which extent findings from rodent OPCs can be translated to the human situation. In studying human OL biology, iPSC‐derived OPCs provide an alternative to studies on scarce fetal or postmortem brain tissue. However, downfalls of iPSC‐derived OPC studies are also evident. Studying aspects such as OPC heterogeneity or functional differences remains difficult as iPSC‐derived OPCs have not experienced the strict developmental programs of migration, proliferation and maturation in the developing brain. Clearly, it should always be taken into account that artificial settings of in vitro cultures are different from the in vivo situation.

### 5.3 Myelin plasticity

Myelin plasticity has been extensively studied in rodents, but is difficult to study in humans. More and more evidence indicates that experience‐dependent neural activity can drive myelination, thereby promoting neural plasticity. To illustrate, physical exercise has been shown to promote differentiation of OPCs and myelination in mice (Simon, Gotz, & Dimou, 2011). In addition, multiple groups have shown that acute differentiation of OPCs is required for complex motor learning in mice. In more detail, motor learning is thought to depend on OPC‐mediated plasticity as neuronal activity of involved motor pathways acutely activates OPC production, differentiation and myelination, thereby improving connectivity (Gibson et al., 2014; McKenzie et al., 2014; Schneider et al., 2016; Xiao et al., 2016). Whether a specific OL subtype is responsible for this type of activity‐dependent myelination is an important question for further investigation. One study showed that specifically immature OLs expressing the stage‐specific marker Enpp6 are increased during a motor task and are required for proper motor learning (Xiao et al. 2016). Furthermore, PDGFR^‐^Sox10^+^ OLs expressing the intracellular calcium channel ITPR2 are increased in number after motor learning, indicating that ITPR2^+^ OLs may also play an important role in this respect (Marques et al., 2016). Whether similar processes of myelin‐mediated motor learning take place in the human CNS is difficult to study at a cellular level. However, imaging studies do provide evidence of experience‐dependent changes in white matter microstructure in response to various types of learning (Zatorre, Fields, & Johansen‐Berg, 2012). To illustrate, several studies revealed that exercise and skill‐learning can improve integrity of white matter tracts (Scholz, Klein, Behrens, & Johansen‐Berg, 2009; Svatkova et al., 2015), indicating that indeed OPC‐mediated white matter plasticity also occurs in humans.

Another example of experience‐dependent myelination was observed in mice; social isolation of juvenile or adult animals negatively affected myelination of the prefrontal cortex (Liu et al., 2012a, 2012b; Makinodan, Rosen, Ito, & Corfas, 2012). Similarly, early social deprivation was associated with reduced white matter integrity of various brain areas, including frontal regions, in human subjects (Eluvathingal et al., 2006; Govindan, Behen, Helder, Makki, & Chugani, 2010). White matter changes after social neglect may therefore contribute to the development of psychiatric disorders in socially deprived individuals (Toritsuka, Makinodan, & Kishimoto, 2015). Together, these findings indicate that activity‐ or experience‐dependent myelination does take place in the human brain. However, whether this type of myelin plasticity requires the production of new OPCs remains unsure considering the stable amount and low turnover rate of OLs as observed by Yeung et al. (2014). To what extent the cellular basis of myelin plasticity in humans is similar to that of rodents remains an important issue to be elucidated.

To summarize, available evidence indicates that the general spatio‐temporal progress of white matter development in rodents closely resembles that of humans, including OPC generation from similar neuroepithelial zones and myelination in a caudal‐to‐rostral manner (Figure 4). By combining findings from experimental rodent studies and imaging studies in humans, it is possible to speculate on the mechanisms underlying phenomena such as activity‐dependent myelination. However, it should be considered that discrepancies between rodents and humans have also been observed in the signaling pathways by which OPC generation, migration, and maturation are regulated. Furthermore, the dynamics by which the existing pool of oligodendrocytes is maintained may differ between rodents and humans. Such possible differences should at least be taken into account when developing novel therapies to enhance (re)myelination.

## 6 IMPLICATIONS FOR PRETERM BIRTH‐RELATED WMI

### 6.1 Preterm birth and diffuse WMI

As discussed above, white matter development is a tightly regulated, intricate process involving many steps. Perinatal insults after preterm birth coincide with the critical window of pre‐OLs populating the white matter (Back et al., 2001) (Figure 5). Hypoxic and/or inflammatory insults are thought to create an unfavorable environment for OLs to fully mature and properly myelinate neuronal axons, thereby dysregulating myelination leading to long‐term impairments in functional outcome (van Tilborg et al., 2016). Whereas cystic WMI (or cPVL) is associated with necrotic/apoptotic cell death and axonal injury (Silbereis, Huang, Back, & Rowitch, 2010), the nowadays predominant diffuse type of perinatal WMI is not necessarily associated with axonal damage. No overt axonal abnormalities were observed in several animal models, but to which extent impaired myelination leads to axonal defects in human diffuse WMI remains unsure (Riddle et al., 2012; van Tilborg et al., 2017). Dysregulated OL development can cause alterations in the microstructure of white matter tracts, which is often observed clinically (Alexandrou et al., 2014; Glass et al., 2008; Mwaniki, Atieno, Lawn, & Newton, 2012; Rutherford et al., 2010; Shankaran et al., 2006; van Vliet, de Kieviet, Oosterlaan, & van Elburg, 2013). However, to which extent myelination in other brain areas such as the neocortex is affected in human children with diffuse WMI, should be further elucidated.

### 6.2 Implications of novel insights in OL biology

#### 6.2.1 Signaling pathways regulating OPC differentiation

Impaired maturation of pre‐OLs causing myelin deficits is an important pathophysiological mechanism of diffuse WMI, making OLs interesting targets for therapeutic strategies. Various pathological mechanisms have been proposed to contribute to impaired OL maturation in diffuse WMI. For instance, inflammatory mediators negatively affect OL development (van Tilborg et al., 2016), but also changes in regulatory pathways such as the Daam2/Wnt/β‐catenin and Notch pathways may contribute to impeded OL maturation in preterm infants (Back, 2017; Fancy et al., 2009, 2011; John et al., 2002; Lee et al., 2015a). Increased activation of JNK signaling in response to perinatal insults has also been implicated in neonatal WMI (Wang, Tu, Huang, & Ho, 2012; Wang et al., 2014). In addition, production of high molecular weight hyaluronan produced by reactive astrocytes has been associated with inhibition of OPC differentiation in MS and perinatal WMI (Back et al., 2005; Buser et al., 2012; Hagen et al., 2014). Inhibiting or reversing the effects of these pathological mediators may be valid therapeutic strategies. However, knowledge on the regulation of healthy OL dynamics may also aid the development of new treatments to combat diffuse WMI in preterm infants. Importantly, the signaling pathways contributing to OPC differentiation may be potential therapeutic targets to overcome the maturational arrest of OLs (see Table 1). Negative regulators of OPC differentiation may be inhibited to allow proper differentiation of OPCs into mature myelinating OLs. For example, BMP4 has been identified as an inhibitor of oligodendrocyte maturation and overexpression of the BMP4 antagonist noggin was shown to protect the white matter from hypoxia‐ischemia‐induced neonatal brain injury in mice (Dizon, Maa, & Kessler, 2011; See et al., 2004). Additionally, activation of the Notch receptor inhibits differentiation of OPCs into mature OLs (Wang et al., 1998). In line, downregulation of Notch signaling by EGF treatment promotes recovery after neonatal WMI in mice (Scafidi et al., 2014). Also, after hypoxia the histone deacetylase (HDAC) Sirt1 promotes proliferation but inhibits differentiation of OPCs. Consequently, Sirt1 inhibition by the HDAC inhibitor sirtinol induces differentiation of OPCs in vitro and may therefore have therapeutic potential for neonatal WMI (Jablonska et al., 2016). Similarly, inhibition of the Wnt pathway by activation of the protein Apcdd1 promotes myelination in hypoxic cerebellar slice cultures indicating potential of Wnt inhibition as a therapeutic intervention for neonatal WMI.

Conversely, stimulating signals that promote OPC differentiation may improve myelination and functional outcome in preterm infants with diffuse WMI. For instance, reduced GABAergic input was shown to contribute to impaired cerebellar OPC maturation in mice with hypoxia‐induced WMI (Zonouzi et al., 2015). Pharmacologically increasing the availability of GABA using antiepileptic drugs reversed the effects of hypoxia and rescued myelination (Zonouzi et al., 2015). However, the exact mechanism requires further investigation as Hamilton et al. (2017) reported a negative effect of endogenous GABA signaling on myelination in cortical organotypic slice cultures. Insulin‐like growth factor (IGF)1 is a trophic factor that has been associated with the positive regulation of OL maturation and IGF1 treatment was shown to protect the white matter in rats with neonatal WMI (Cai, Fan, Lin, Pang, & Rhodes, 2011; Pang et al., 2010) (Table 1).

Of note, treatments that have been shown to promote remyelination in animal models of multiple sclerosis may also have beneficial effects on myelination in the developing brain and are therefore of great interest as potential treatments for perinatal WMI (Franklin, 2015). For example, anti‐inflammatory compounds such as activin‐A, and activation of retinoid X receptor γ (RXR‐γ) have been shown to promote remyelination, and may therefore also promote myelination during development (Huang et al., 2011; Miron et al., 2013) (Table 1). Additional examples of pathways implicated in the regulation of OPC differentiation of which intervention may enhance myelination in WMI are listed in Table 1.

#### 6.2.2 OPC–vascular interactions

As discussed in “OPC migration,” experimental data indicate that OPCs interact closely with the brain vasculature. Electron microscopy of human postmortem brain tissue revealed that similar interactions take place in the human brain (Maki et al., 2015). From these findings, the question rises whether interactions between OPCs and blood vessels may contribute to neonatal WMI. Interestingly, it was demonstrated that vascular OPCs may play a dual role in WMI in adult mice. Initially, OPCs can contribute to injury by inducing blood–brain barrier leakage, but later they can contribute to vascular remodeling during recovery (Pham et al., 2012; Seo et al., 2013). Whether similar mechanisms play a role in WMI in the developing brain remains to be investigated, but the close interaction between OPCs and the vasculature indicates that specifically these OPCs may be targeted through systemic intervention.

#### 6.2.3 Timing of insults

Other recent insights in the biology underlying white matter development also have implications for understanding the pathophysiology of WMI in preterm infants. For example, the developmental stage during which preterm infants are exposed to perinatal insults ranges from early maternal infections (in utero) to postnatal episodes of inflammation and/or hypoxia (Chau et al., 2012, 2009; Fyfe, Yiallourou, Wong, & Horne, 2014; Glass et al., 2008). Considering the different developmental processes taking place in the white matter at these distinct times, it is possible that the timing of an insult impacts the extent and localization of WMI, as well as related outcome (Back et al., 2001; Semple et al., 2013). For instance, different migratory streams of OPCs may be affected by early or late inflammatory or respiratory insults. Combining clinical data of preterm infants with imaging data and follow‐up data may reveal the role of the timing of certain insults on the extent and localization of WMI.

#### 6.2.4 Origin of OPCs

As discussed in “Similarities between rodent and human myelination,” most evidence indicates that the pool of OPCs in the human brain develops similar to that of rodents, with an initial dorsal stream of OPCs that, over time, shifts to a more ventral stream. Crawford et al. (2016) showed in the rodent spinal cord that OPCs from different origins have variable susceptibility. Whether OPCs from distinct developmental origins or OPCs with distinct transcriptional profiles are differentially susceptible to perinatal insults is an interesting question to further explore in future research. In case certain OPCs are more susceptible than others, targeting receptors specifically expressed by such vulnerable OPC subsets may be an interesting therapeutic strategy.

#### 6.2.5 Compensatory mechanisms

As explained in “OPC generation” and “Classification based on expression of receptors/ion channels,” rodent studies revealed that myelination is a relatively robust process with an abundance of OPCs being generated, creating a pool of “backup” OPCs that can start differentiating in case of injury to surrounding OLs (Kessaris et al., 2006; Vigano et al. 2016). Whether similar compensatory mechanisms take place in the human brain, and whether such mechanisms are active in preterm infants with WMI, are interesting questions to further explore. In case compensatory mechanisms are at play, boosting such regenerative processes may promote the recovery of neonatal WMI.

#### 6.2.6 Myelin plasticity in WMI

Animal and human studies have shown that neural activity stimulates OPC differentiation and myelination during motor learning (Scholz et al., 2009; Svatkova et al., 2015; Zatorre et al., 2012). With this in mind, preterm infants may benefit from neural stimulation to promote white matter development. Physical therapy or tactile/auditive stimulation may have beneficial effects and may complement other therapeutic strategies that are currently being investigated, such as anti‐inflammatory treatments or stem cell therapy. Indeed, several studies revealed that music has beneficial effects on preterm infants in terms of cortisol levels, heart rate, and pain (Qiu et al., 2017; Schwilling et al., 2015), but due to contradictory results more research is required to accurately assess the effects of sensory stimulation on white matter development and outcome in preterm infants (Bieleninik, Ghetti, & Gold, 2016; Pineda et al., 2017).

In sum, knowledge on the developmental processes underlying healthy white matter development is crucial to come up with novel treatment options for when proper myelination is hampered. More research into the different functionalities and vulnerabilities of specific OPC subsets is essential to gain more insight into white matter development. The different aspects of OL lineage development and dynamics should be taken into account when exploring the pathophysiological mechanisms underlying WMI and pursuing novel therapies for WMI.

## 7 CONCLUDING REMARKS

Over the past years, experimental research, mostly in rodents, provided much insight in novel concepts regarding OL development and myelination. As depicted in Figure 4, gross white matter development in humans shows high resemblance to that of rodents (Jakovcevski & Zecevic, 2005a, 2005b; Rakic & Zecevic, 2003). However, it should be taken into account that some differences between rodent and human OLs have been reported at a cellular level (Filipovic & Zecevic, 2008; Wilson et al., 2003).

During development, OPCs are generated over the course of multiple waves, originating from distinct brain regions. Exactly how the spatial and temporal origins of OPCs determine their functionality remains to be further investigated, but evidence suggests that OPCs converge into a single pool of cells that disperse throughout the CNS and adapt their gene expression profile to the needs of their environment (Marques et al., 2016). Most OPCs eventually differentiate into mature OLs to myelinate neuronal axons. Rodent studies revealed that myelination is a highly dynamic process with an excess of OPCs being generated in order to compensate for possible injury to developing OLs (Kessaris et al., 2006; Vigano et al., 2016). Furthermore, OPCs can contribute to neural plasticity by inducing activity‐dependent myelination, which is required for motor learning (Marques et al., 2016; McKenzie et al., 2014). These insights should be taken into account in order to understand the pathophysiological mechanisms underlying WMI in preterm infants. To date, the precise mechanisms underlying impaired maturation of OLs in perinatal WMI are not fully understood. Therefore, it is crucial to perform more research into white matter development under normal and pathological situations in order to develop novel OL‐targeted therapies to combat WMI and its severe consequences in preterm infants.
